# Respiratory Syncytial Virus Positivity Rate and Clinical Characteristics Amongst Children Under 5 Years of Age at the Emergency and Outpatient Settings in Jordan: A Cross-Sectional Study

**DOI:** 10.3390/v18010133

**Published:** 2026-01-20

**Authors:** Munir Abu-Helalah, Samah F. Al-Shatnawi, Mohammad Abu Lubad, Enas Al-Zayadneh, Mohammad Al-Hanaktah, Mea’ad Harahsheh, Montaha Al-Iede, Ruba Yousef, Mai Ababneh, Toqa AlZubi, Suad Abu Khousa, Mohammad Al Tamimi, Simon B. Drysdale

**Affiliations:** 1 Department of Family and Community Medicine, Faculty of Medicine, University of Jordan, Amman 11942, Jordan; 2 Public Health Institute, University of Jordan, Amman 11942, Jordan; 3 Department of Clinical Pharmacy, Faculty of Pharmacy, Jordan University of Science and Technology, Irbid 22110, Jordan; sfshatnawi@just.edu.jo; 4 Faculty of Medicine, Mutah University, Karak 61710, Jordan; abu_lubbad@mutah.edu.jo; 5 Department of Pediatrics, Faculty of Medicine, University of Jordan, Amman 11942, Jordan; e.alzayadneh@ju.edu.jo (E.A.-Z.); m.al-iede@ju.edu.jo (M.A.-I.); 6 Faculty of Medicine, University of Jordan, Amman 11942, Jordan; malhanaktah49@gmail.com (M.A.-H.); suadabukhousa@gmail.com (S.A.K.); 7 School of Pharmacy, University of Jordan, Amman 11942, Jordan; mya9220222@ju.edu.jo; 8 Medical Department, MENA Center for Research and Development, Amman 11942, Jordan; ruba.yousef1995@gmail.com; 9 MENA Center for Research and Development, Amman 11942, Jordan; mai.ababneh97@gmail.com (M.A.); toqaalzoubi66@gmail.com (T.A.); 10 Independent Researcher, Amman 11942, Jordan; 11 Oxford Vaccine Group, Department of Pediatrics, University of Oxford, Oxford OX3 7LE, UK; simon.drysdale@paediatrics.ox.ac.uk; 12 The NIHR Oxford Biomedical Research Centre, Oxford OX3 9DU, UK

**Keywords:** RSV, clinical, epidemiological, outpatients, emergency, pediatrics, Jordan

## Abstract

Background: Acute viral respiratory infections are a major cause of morbidity among young children, with respiratory syncytial virus (RSV) being the leading pathogen. In Jordan and globally, most RSV research has focused on hospitalized patients, while data from emergency departments (EDs) and outpatient settings remain limited. Methods: This cross-sectional study was conducted at two major Jordanian hospitals between November 2022 and March 2023. Children under five years of age presenting to EDs or outpatient clinics with symptoms of acute respiratory infection were enrolled. Nasopharyngeal specimens were tested for RSV, and subtypes (RSV-A and RSV-B) were identified using multiplex RT-PCR. Results: Of 229 enrolled children, 92 (40.2%) tested positive for RSV, with RSV-B accounting for 81.5% of positive cases. RSV positivity was higher in ED presentations than in outpatient clinics (46% vs. 35%). Wheezing (72.8% vs. 39.4%, *p* < 0.001) and dyspnea (33.7% vs. 14.6%, *p* = 0.001) were significantly more frequent among RSV-positive patients. Independent predictors of RSV positivity included non-referred outpatient visits (OR = 15.26), non-referred ED visits (OR = 42.93), younger age, and prior systemic steroid use. Conclusions: RSV poses a substantial burden in outpatient and ED settings. Identified demographic and clinical predictors may help target high-risk groups for future preventive interventions.

## 1. Introduction

In children, acute respiratory infections, particularly viral infections, are a significant source of morbidity and mortality [1]. Respiratory syncytial virus (RSV) is the primary cause of acute respiratory infections in children, primarily causing bronchiolitis and pneumonia [2].

Estimates suggest that 22% of global cases of acute respiratory tract infections occur in low-income countries. Moreover, data have revealed that RSV is responsible for around 33 million acute respiratory infections (ARIs), resulting in 3.6 million hospitalizations for severe cases and approximately 100,000 deaths among children under 5 years old [3,4].

Recent data from Jordan indicate that RSV imposes a significant burden on the country. A recent study included 1000 children younger than 5 years from inpatient sites in Jordan, with a mean age of 17.10 months (SD = 16.57). The positivity rate for RSV infections was 50.6% [5].

Limited data are available at the regional and international levels on the positivity rates and health and economic impact of RSV for patients attending Emergency Departments (EDs) and outpatient clinics [6]. Most studies focus on the hospital and community burden of RSV. Interestingly, a recent systematic review by Heemskerk et al. found that the incidence rates of RSV infection in the emergency department (ED) setting were similar to those in the community setting, and that primary care incidence estimates were higher [7]. A recent study revealed that around 19% of children younger than 4 years of age presenting with influenza-like illness at emergency departments in New York City tested positive for RSV [8]. The burden of RSV infections at the ED and outpatient clinics has not been assessed in Jordan and the Middle East. We, therefore, aimed to determine RSV positivity and outcomes in these settings.

## 2. Materials and Methods

### 2.1. Study Design

This cross-sectional study involved 2 study centers: (1) Princess Rahma Hospital for Children, Irbid; (2) Jordan University Hospital, Amman, between November 2022 and March 2023. The former hospital is the only pediatric hospital in northern Jordan and serves the largest city in the north and the third-largest city in Jordan. This hospital has 163 beds and 42 NICU incubators. Jordan University Hospital (JUH) is located in Amman, Jordan’s capital. JUH has 550 beds, with the possibility of expanding to 600 beds along with 24 surgical operating rooms and eight specialist intensive care units. JUH treats over 500,000 patients in its outpatient clinics, performs 25,000 surgeries, and sees 94,000 emergency room cases each year. Patients at these hospitals are generally representative of Jordanian pediatric patients. Furthermore, to avoid convenience sampling, data were collected on different dates and times, including night shifts and weekends.

The primary outcome of this study was to assess the proportion of RSV-positive children under 5 years of age in emergency departments (EDs) and outpatient settings in Jordan. Secondary outcomes included the epidemiological and clinical characteristics of RSV infections at the ED and the outpatient department at the study sites. The analytical component of this cross-sectional study included regression analysis to identify predictors of RSV positivity. These outcomes were compared between RSV-positive and RSV-negative cases and between RSV-A- and RSV-B-positive patients.

### 2.2. Study Population

#### 2.2.1. Inclusion Criteria

Patients who permanently resided in the study areas and visited the outpatient clinic and emergency department at the study sites with an acute respiratory infection, according to the definition and criteria below.

#### 2.2.2. Case Definition [9]

Diagnosis of acute respiratory infection, which was defined as “an illness presenting with one or more of the following symptoms for less than 7 days: Fever, cough, earache, nasal congestion, rhinorrhea, sore throat, vomiting after coughing, crackles, and labored, rapid or shallow breathing”.

Our sample included children younger than 5 years of age with acute respiratory infection, which ensured high inclusion, according to the below criteria, and who were treated as outpatients or presented to the emergency departments at the study sites with the following:

(1) At least one sign of an acute infection (temperature ≥38 °C or <35.5 °C, abnormal white blood cell [WBC] count, or abnormal differential).
(2) At least one of the following respiratory signs or symptoms for less than 7 days: tachypnea, cyanosis, cough, sputum production, pleuritic chest pain, hemoptysis, dyspnea, sore throat, runny nose, grunting, wheeze, apnea, and difficulty breathing.

Patient recruitment occurred on weekdays and continued on weekends and holidays to support diversity in access to enrollment and to ensure representativeness of the sample.

#### 2.2.3. Exclusion Criteria

(1) Not a permanent resident of Jordan.
(2) Patients discharged from the hospital who returned within 2 weeks with symptoms that did not resolve for 48 h.

#### 2.2.4. Sample Collection and Processing

- Nasopharyngeal (NP) specimens were collected from each patient who met the inclusion criteria and consented to this study. An NP swab was taken, and then a Multiplex viral RT-PCR was performed on each nasopharyngeal specimen.
- PCR was used to diagnose cases of RSV at the included sites.
- RSV-positive samples were further analyzed via genotyping for RSV-A and RSV-B.

### 2.3. Microbiology

#### Sample Collection and Transport

Flocked swabs with plastic shafts were used to collect nasopharyngeal specimens from each patient who met the inclusion criteria. The swabs were then inserted into sterile viral transport media (VTM) and immediately placed on refrigerant gel packs or held at 4 °C before transport to the laboratory on the day of collection. Upon arrival at the laboratory, the specimens were immediately processed. Nucleic acid was extracted from the specimens and stored at −80 °C for subsequent analysis to identify the target viruses.

Multiplex RT-PCR (TaqPath™ Combo Kit; Thermo Fisher, Waltham, MA, USA) was performed on each nasopharyngeal specimen to test for respiratory syncytial virus (RSV), as specified by the manufacturer, using a QuantStudio 5 (Applied Biosystems, Foster, CA, USA). RSV genotypes A and B were detected using the VIASURE Real-Time Detection Kit (CerTest Biotec S.L., Zaragoza, Spain). The RT-PCR cycles were as follows: 1 cycle for reverse transcription for 15 min at 45 °C, Initial denaturation for 2 min at 95 °C, 45 cycles of denaturation for 10 s at 95 °C, and 45 cycles of Annealing/Extension for 50 s at 60 °C [10,11].

### 2.4. Power/Sample Size

We aimed to enroll 225 subjects who matched the above clinical criteria. Based on previous studies, we anticipated that approximately 19% of these subjects would be positive for RSV [8]. Therefore, we expected 20% (*p*) of the subjects to be positive for RSV when calculating the sample size. We targeted a two-sided 95% confidence interval with precision (half-width) *E* = 0.05 for the primary estimate.

$$n=\frac{Z_{0.975}^{2}\;p(1-p)}{E^{2}}$$

### 2.5. Statistical Methods

SPSS version 28 was used to analyze the data. Descriptive statistics (Student’s *t*-test and chi-squared test) were used to analyze and compare categorical variables, including demographic characteristics, patient characteristics, risk factors, pre-hospital antibiotic use, and vaccination details. Forward multi-stage Logistic regression analysis was used to identify predictors of RSV positivity and complications.

### 2.6. Case Report Form (CRF)

Each study participant had a unique case ID throughout the study. The first part of the form included the study’s inclusion criteria, as described above. The parents/guardians of eligible patients were asked whether they consented to participate in this study after being informed of the study details. All were reassured that participation was voluntary and would not affect the service provided.

The interview forms included five sections:

1. Background, demographic, and societal data for patients and parents: Sex, age, parents’ ages and educational statuses, special diet/milk, number of people in the household with age groups, patients or siblings attending a kindergarten, and parents or siblings with a history of asthma or eczema. Furthermore, a detailed smoking history was obtained, including the mother’s history of smoking during pregnancy; the father’s, mother’s, or other household members’ smoking (inside or outside the home); and the number of cigarettes per day or waterpipe per week.
2. Medical history, including birth history, existing medical conditions, and current regular medications: weight, height, birth weight, gestational age at birth, method of delivery, meconium-stained liquor, neonatal ICU admission and ventilation status, surfactant, breast feeding, asthma, cystic fibrosis, bronchopulmonary dysplasia, neuromuscular disease, congenital heart disease, other congenital disease, immunodeficiency, eczema (atopy), and other comorbidities. Any previous hospital admissions with the diagnosis, duration, and dates were included. Vaccine status for pneumococcus, influenza, and SARS-CoV-2 was obtained for the patient, mother, father, and siblings. Regular medications and their indications. Medical history of RSV treatments during the current admission or previous admissions: Ribavirin, monoclonal antibodies, and RSV immunoglobulin.
3. Presenting symptoms and signs: Symptoms and their duration before visit to the Emergency department or to the outpatient clinic were included. Other clinical manifestations included cardiovascular manifestations, dehydration, wheezing, cyanosis, reduced activity level, hypoxia (SpO2 < 92%), tachypnea, pneumothorax/atelectasis, apnea >10 s, subcostal/intercostal retractions, and nasal flaring.
4. Laboratory findings, including WBC and differential, PCR results, chest X-ray findings, and pharyngeal swabs.
5. Healthcare services utilized before hospitalization at public or private hospitals and clinics, and the financial cost of the provided care.

All medications used during the outpatient or emergency department stay, including the name, route, dose, frequency, number of days of admission, and final cost per medication, were recorded for each patient. This included all prescribed discharge medications.

6. Outcomes of the visit: admission, discharge, or follow-up visit were assessed.

## 3. Results

### 3.1. Patient Demographics, Clinical, and Financial Data

A total of 347 patients were screened for study inclusion, with 162 (46.7%) presenting through the Emergency Department (ED) and 138 (39.8%) through Outpatient (OP) services. Among OP visits, the majority were new patients (n = 118, 85.5%), whereas returning patients from the ED accounted for 14.5% (n = 20). Similarly, new patients accounted for a larger proportion of ED visits (n = 111, 68.5%) than returning patients (n = 51, 31.5%). Overall, new patients accounted for 66.0% (n = 229) of the total cohort, whereas returning patients accounted for 34.0% (n = 118). Six patients were excluded because they were discharged from the hospital and returned within 2 weeks with persistent symptoms.

**Figure 1:**
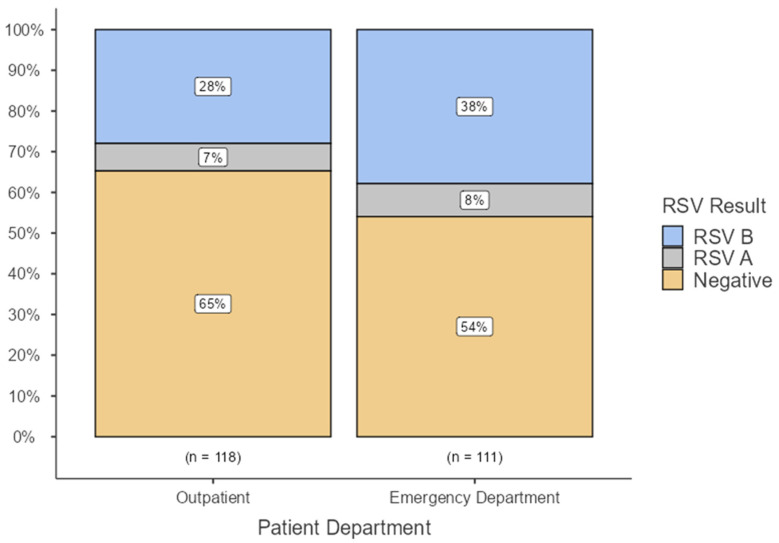
RSV Positivity and subtype by route of presentation.

In total, 229 participants were recruited from the emergency department and outpatient settings, ensuring representation of the targeted population (Table 1). Among these, 92 patients tested positive for RSV, with a positivity rate of 40.2% and an average age of 17.47 months (Table 1). RSV-B represented 81.5% of RSV-positive cases (46% of all sample), while RSV-A represented the remaining 18.5% of them (35% of the total sample). The positivity rate among patients who presented to the emergency department was 46% (8% RSV A and 38% RSV B). These rates are higher than those reported from outpatient clinics, with 35% (7% RSV A and 28% RSV B). RSV-positive cases had higher wheezing (72.8% vs. 39.4%, *p* < 0.001) and dyspnea (33.7% vs. 14.6%, *p* = 0.001) (Figure 1). Independent predictors included non-referred outpatient (OR = 15.26, *p* < 0.001) or non-referred ED visits (OR = 42.93, *p* < 0.001), younger age (OR = 0.964 per month, *p* < 0.001), and prior systemic steroid use (OR = 0.160, *p* = 0.021) (Table 1).

The majority of RSV-positive cases were male (57.60%), younger than 2 years (74%), and from the northern region (65.5%). Additionally, RSV-B infection predominated over RSV-A within the northern region (Table S1) (89.3% vs. 64.7%, *p* < 0.05). RSV-positive patients exhibited significantly higher rates of wheezing (72.8% vs. 39.4%; *p* < 0.0005), difficulty breathing (33.7% vs. 14.6%; *p* = 0.001), pleuritic chest pain (5.4% vs. 0%; *p* = 0.006), and grunting (7.6% vs. 1.5%; *p* = 0.019), compared to RSV-negative patients. Conversely, sore throat (27% vs. 9.8%; *p* = 0.001) and sputum production (46.7% vs. 19.6%; *p* = 0.000) were more prevalent among RSV-negative patients (Table 2).

RSV positivity was associated with factors such as lack of surfactant administration (*p* = 0.002), presence of siblings younger than the age of 5 in the household (*p* = 0.006), and lower parental education levels (*p* < 0.001) (Table 3). Parents with a history of asthma and atopic eczema were less likely to have RSV-positive children (*p* < 0.05). Surprisingly, favorable rates were much higher among exclusively breast-fed children or those with mixed breast-feeding and formula compared to children with no history of breast-feeding (*p* = 0.014). Children of non-smoking parents had a lower positivity rate than children of smoking parents (*p* = 0.002). However, these two factors were not statistically significant in the regression analysis (see below), suggesting the presence of additional confounding factors (Table 3).

RSV-positive children demonstrated markedly higher frequencies of several clinical findings compared to RSV-negative cases, with chest X-ray infiltrates being significantly more common among RSV-positive patients (77.17% vs. 38.69%, *p* < 0.001), indicating a substantially greater burden of radiological involvement. Abnormal blood routine counts were also significantly associated with RSV positivity, with both low (<4.0) and high (>10.0) values observed more frequently in RSV-positive patients (30.43% and 30.43%, respectively) than in RSV-negative patients (14.60% and 16.79%, respectively; *p* < 0.001). RSV-positive children had a significantly higher prevalence of wheezing (73.91% vs. 43.07%, *p* < 0.001), whereas other clinical manifestations were paradoxically more common among RSV-negative patients (30.66% vs. 9.78%, *p* < 0.001). Tachypnea was also significantly more frequent in RSV-negative children (25.55% vs. 14.13%, *p* = 0.044). No significant differences were observed across other clinical signs, including cardiovascular findings, activity level, apnea, dehydration, hypoxia, retractions, cyanosis, co-infections, acid–base status, or oxygen-ventilation requirements (all *p* > 0.05). Overall, the most prominent distinguishing features of RSV infection were the substantially increased rates of chest X-ray infiltrates, abnormal blood counts, and wheezing, underscoring their diagnostic relevance in RSV-positive pediatric presentations (Table 4).

Further analysis was performed to compare patients positive for RSV-A to those positive for RSV-B. There was no statistically significant difference between RSV-A and RSV-B in terms of the presence of clinical features. The only statistically significant differences were reported for the duration of symptoms, such as Post-Tussive Vomiting and chrackels, which had shorter durations in RSV-B-positive patients (Table 5).

### 3.2. Predictors for RSV Positivity and Cost in the Emergency and Outpatient Departments

Binary logistic regression analysis was conducted to identify factors associated with receiving a positive RSV result across all age groups. This included demographic variables, medical history, and clinical symptoms. The results are summarized in Table 6. Children who visited outpatient clinics or EDs without referral had significantly higher odds of RSV positivity than those referred to the ED (OR = 15.26 and 42.93, respectively; *p* < 0.001). Younger age was also significantly associated with higher RSV positivity (OR = 0.964 per month, *p* < 0.001). The use of systemic steroids before presentation was associated with significantly lower odds of RSV positivity (OR = 0.160, *p* = 0.021).

## 4. Discussion

This study highlights the significant burden of RSV infections, with 40.2% positivity rates among children younger than the age of 5 presenting with respiratory symptoms at emergency departments (44%) and outpatient clinics (35%). These figures from Jordan are close to reported data from Western countries on RSV positivity at outpatient clinics or emergency departments following the COVID-19 pandemic [12,13].

One multi-center cross-sectional study in Jordan (including Amman) from November 2022 to April 2023 found that 50.6% of hospitalized children under 5 years with respiratory symptoms were RSV-positive. This is consistent with previous studies, which report higher rates among inpatients than among outpatients or emergency department patients. A US study revealed that RSV is associated with a lower percentage of general office (outpatient) visits for acute respiratory infections compared to emergency department and inpatient visits (e.g., 15% of office visits vs. 18% of ED visits vs. 20% of hospitalizations in one study) [14].

### 4.1. RSV Positivity

We found a high RSV positivity rate among participants in this study. These figures are consistent with post-COVID data from Europe and Taiwan and exceed global pre-COVID-19 figures. A large prospective cohort study from Europe was conducted to examine RSV positivity at primary care settings in Belgium, Italy, Spain, the Netherlands, and the UK during the RSV seasons of 2020–2021 (UK only; from 1 January 2021), 2021–2022, and 2022–2023. Children aged younger than 5 years presenting to their general practitioner or primary care paediatrician with symptoms of an acute respiratory tract infection were included. Among 3414 tested children, 1124 (32.9%; 95% CI 31.3–34.5) tested positive for RSV [12]. This figure is very close to our 35% result across our outpatient clinics. Additionally, emergency department visits were high during the COVID-19 pandemic, attributable to a post-pandemic surge in respiratory infections. A study from Northern Taiwan reported that in 2022 and 2023, a large proportion of RSV tests ordered in the emergency department for children with respiratory symptoms were positive (59.2% and 74.5%, respectively) [13].

Before the pandemic, most figures were in the 10–30% range. Studies from the USA on RSV positivity at the ED and outpatient settings showed lower positivity rates than reported in our study, with rates between 18% and 28% [14,15,16,17]. However, RSV positivity rates were high in Jordan even before the COVID-19 pandemic, underscoring the importance of RSV prevention. Our figures are consistent with findings from a Jordanian study conducted between 2010 and 2013 at a single center, which found that 44% of 3168 children under two years old admitted to the hospital tested positive for RSV [18]. This highlights the significant burden of RSV among young children in Jordan, where a substantial proportion of hospitalizations for respiratory illness were associated with RSV infection. Our study provides critical data on RSV’s impact within this population, reinforcing the need for preventive measures and early interventions to reduce RSV-associated morbidity and hospitalization.

Further evidence comes from a recent systematic review [4] that assessed RSV and calculated a global incidence rate of 53.1 (39.2–72.0) per 1000 children under 5 years old annually. This systematic analysis estimated approximately 57,798 (42,467–78,331) annual episodes of RSV-associated acute lower respiratory tract infections among young children [4]. Such figures underscore RSV’s substantial impact on public health in Jordan, aligning with findings from neighbouring countries in the Middle East and North Africa (MENA) region [19]. The incidence rates in Jordan mirror those reported in countries with similar demographic characteristics, healthcare access, and environmental factors, suggesting that the burden of RSV may be similarly distributed across comparable settings. This consistent incidence across Jordan and similar regional contexts emphasizes the value of targeted RSV prevention strategies, especially among vulnerable populations such as infants and young children. Recognizing RSV as a leading cause of acute lower respiratory tract infections has important implications for healthcare policy, including the prioritization of RSV surveillance, vaccination, and treatment efforts. These data further suggest that RSV prevention and vaccination programs could significantly reduce RSV-related hospitalizations and improve child health outcomes in Jordan and throughout the MENA region, where RSV remains a leading cause of respiratory disease in young children.

The high positivity rates at the emergency department and outpatient settings in Jordan emphasize the role of prevention and surveillance for RSV in children with respiratory symptoms in these settings. This is supported by our previous work on RSV burden in inpatient settings, which revealed a 51% positivity rate among children hospitalized with respiratory symptoms at 4 representative hospitals in Jordan. Therefore, it is recommended to routinely test infants for RSV in Emergency departments and those in inpatient wards, and to urgently develop local guidelines in Jordan for immunoprophylaxis of all neonates or at least high risk groups, according to available resources. This trend of mass prophylaxis for all neonates has been shown to have cost-effective outcomes either through maternal vaccination or infant monoclonal antibodies. Studies have shown that universal monoclonal antibody use can prevent approximately 75–85% of RSV-related hospitalizations and up to 80% of RSV-related deaths in infants during their first season [20,21]. However, a recent report highlighted that although a monoclonal antibody provides substantial health benefits, implementation is restricted by high acquisition costs and the lack of comprehensive global funding for mAbs compared with vaccines [22]. Therefore, in countries with limited resources such as Jordan, initial recommendations may be to offer a monoclonal antibody for RSV prevention to only high-risk groups according to local data, with the potential for mass prophylaxis if sufficient resources are available.

### 4.2. RSV Subgroups

We showed that a significant proportion of RSV cases, particularly in northern Jordan, were attributable to RSV-B. This finding aligns with the global trend observed during the 2022/23 RSV season, when RSV-B emerged as the dominant circulating subgroup both locally, according to unpublished data, and globally [23,24,25,26]. The trend prior to the COVID-19 pandemic was different, with dominance of RSV-A. A study from the USA published in 2013 revealed that RSV-B accounted for 35% of RSV-positive infants seen in the Emergency Department and was associated with a lower hospitalization rate and ICU admission rate compared with infants with RSV-A [27]. Studies across the globe reported a similar dominance of RSV-B during this period, confirming that our findings are consistent with global epidemiological trends [23,24,25,26]. Our study’s outcomes reinforce the generalisability of our data to comparable settings within Jordan and beyond, particularly during the 2022/23 RSV season. Since RSV typically exhibits biennial or seasonal fluctuations, the dominance of RSV-B in this season is consistent with a broader trend that may be cyclical or influenced by environmental and social factors, such as climate or population density. Such epidemiological insights are valuable for informing local and regional RSV prevention strategies, as understanding prevalent RSV subgroups enables targeted interventions and preparation for future outbreaks. As RSV vaccines and monoclonal antibodies are increasingly developed and used, identifying circulating subgroups is essential for monitoring vaccine efficacy, strain-specific immunity, and the emergence of escape mutants. Future larger studies would provide more comprehensive data in this regard.

### 4.3. Clinical Features of RSV Cases

Infants and children in our cohort exhibited the classical features of RSV infection, including respiratory distress, wheezing, fever, cyanosis, and apnea. We noted that RSV-positive patients had significantly higher rates of wheezing, difficulty breathing, pleuritic chest pain, and grunting than RSV-negative patients. In contrast, sore throat and sputum production were more common in RSV-negative patients. We noted that a very high proportion of our cohort in both the RSV-positive and RSV-negative groups (>96% overall) had a documented fever. This is higher than other studies [28,29] investigating infants and children with RSV and may reflect the different populations recruited. We included many more older children (mean age of RSV cases was 17 months), who are more likely to have a fever with RSV than young infants [30].

We noted that breathlessness and post-tussive vomiting were more common in RSV-A cases than in RSV-B cases; however, there are no specific clinical features that reliably differentiate infants with RSV-A from RSV-B. There were similar lengths of total hospital and ICU admissions and total consumable costs for RSV-A and RSV-B cases. The findings suggest that RSV-A and RSV-B positivity may not have a substantial differential impact on clinical features. A recent review [31] concluded that although there is a tendency for higher disease severity to be attributed to RSV-A, no consensus could be reached as to whether either subgroup caused more severe outcomes. We previously found that infants in both subgroups had similar clinical features, disease severity, and viral dynamics [30].

### 4.4. Predictors for RSV Disease

This study demonstrates that children without clinical features such as nasal obstruction or respiratory crackles were more likely to test positive for RSV. This may be partially explained by the age differences observed in our cohort; children who tested positive for RSV were, on average, 12 months younger than those who tested negative. Older children presenting to healthcare are less likely to present with subtler symptoms of RSV, potentially due to differences in immune responses and respiratory physiology at this age. These findings emphasize the importance of considering age and less obvious symptoms when diagnosing RSV in older children. An additional finding of this study is an association between higher COVID-19 vaccine uptake among participants’ families and a lower likelihood of RSV positivity. Moreover, participants who were not vaccinated against COVID-19 or influenza were more likely to test positive for RSV. These findings are likely related to a combination of socio-economic factors: parents who are more likely to be vaccinated may have fewer other risk factors for infant RSV infection, and to the availability and local recommendations for COVID-19 and influenza vaccines in younger children. Although an RSV-specific vaccine was not widely available for young children at the time of this study, we highlight the potential broader benefits of high vaccine uptake in reducing RSV incidence, even in the absence of an RSV-specific vaccine.

In line with previous studies [32,33], we found that children who did not receive surfactant therapy—used here as a proxy for significant prematurity—were at a higher risk for RSV positivity. Children born prematurely often have underdeveloped respiratory and immune systems, increasing their susceptibility to RSV and other respiratory infections. Additionally, we observed that lower levels of parental education were associated with higher RSV positivity rates. This could be linked to limited access to healthcare resources or health literacy disparities, which might impact awareness of preventive measures and early signs of infection. These findings suggest that socioeconomic and medical risk factors can play a significant role in RSV susceptibility, supporting targeted interventions for high-risk groups.

Exclusive breastfeeding was surprisingly associated with higher rates of RSV positivity. This could be justified by a confounding factor, such as parental education, because it lost statistical significance in the logistic regression analysis. While exclusive breastfeeding is generally protective against various infections, including RSV, a limited number of studies have suggested that exclusive breastfeeding at the onset of symptoms might be associated with a slightly increased risk of viral respiratory infections, potentially due to closer contact with the mother. However, this is not a universally accepted finding, and other studies show that breastfeeding, including exclusive breastfeeding, is protective against RSV bronchiolitis [34].

### 4.5. Strengths

This study included a large sample of children prospectively recruited from two distinct regions of Jordan, representing the diversity of the country’s population. By selecting participants from various clinical settings—ranging from outpatient cases with mild symptoms to severe cases requiring hospitalization—the study provides a comprehensive picture of RSV’s impact across different disease severities. This broad inclusion enhances the generalizability of our findings to other countries with similar healthcare infrastructures and epidemiological profiles, making our data highly relevant to public health planning beyond Jordan. For RSV testing, we used real-time reverse-transcriptase polymerase chain reaction (RT-PCR), a method known for its high sensitivity and specificity in detecting RSV RNA. This molecular technique significantly reduces the likelihood of misclassification, ensuring that RSV cases are accurately identified. Given RSV’s potential for causing asymptomatic or mild infections, the precision of RT-PCR testing helps capture an accurate representation of RSV prevalence, even in less severe cases that might otherwise go undiagnosed. This accuracy is essential for generating reliable epidemiological data, guiding appropriate interventions, and informing the development of targeted RSV prevention programs. Overall, the methodological rigor of this study supports the reliability and applicability of its findings, providing a robust basis for future RSV research and healthcare initiatives.

### 4.6. Limitations

This study emphasizes the importance of studying RSV positivity in the emergency department and outpatient settings to obtain a more comprehensive picture of disease burden. However, it has some limitations. It was conducted at two sites. Princess Rahma Hospital is the only pediatrics hospital in the North of Jordan and Jordan University Hospital is a large tertiary hospital. The high positivity rate at these sites reflects the situation in Jordan, but more work is needed to include other sites. Another limitation is that the study was conducted only during the RSV season. This is primarily due to budget constraints and the fact that, based on previous studies, the RSV season in Jordan occurs during our study period. Variables such as breastfeeding, parental education, and parental smoking may not have reached statistical significance in the regression model due to the small number of RSV-positive cases. However, they were significant in the bivariate analysis. Risk factors such as birth history are subject to recall bias, particularly when items such as surfactant for children not born at Ministry of Health hospitals or Jordan University Hospital are involved, where data are available and electronic. Additionally, we did not perform genetic sequencing of the RSV-positive samples and identified only serogroups A and B. The small number of cases with positive RSV-A and RSV-B has limited the ability to compare them. This study was conducted during the 2022/2023 RSV season, a time still impacted by the residual effects of the COVID-19 pandemic. For older children in our research—many of whom were born during the pandemic—their immune responses to RSV might differ due to altered early-life exposure to common respiratory viruses. Changes in social interactions, masking, and lockdowns could have affected immunity development and RSV susceptibility. Nonetheless, the main innovation of this study lies in filling the data gap in Jordan’s emergency and outpatient settings. Future studies may need to account for these factors to fully understand RSV epidemiology in the post-pandemic context.

## 5. Conclusions

This is one of the few regional and global studies examining the burden of RSV infections in outpatient and emergency department settings. These RSV positivity rates and clinical manifestations are consistent with post-COVID-19 data on RSV’s significant burden in outpatient and ED settings. This study also identified demographic, socioeconomic, and medical predictors of RSV positivity and RSV complications. These groups could be targeted in future interventions as high-risk groups for RSV burden. This study will therefore contribute to ongoing work on the burden and prevention of RSV infections in Jordan. This study also highlights the need for routine RSV testing, at least during RSV season, in emergency departments and outpatient settings for children aged 5 years or younger presenting with respiratory symptoms. In countries with limited resources, such as Jordan, it might be recommended to develop national guidelines for offering monoclonal antibodies for RSV prevention to high-risk groups according to local data with the potential of mass prophylaxis if sufficient resources are available.

## Figures and Tables

**Table 1:** Demographic factors associated with RSV results in the emergency and outpatient departments.

Variable	RSV Negative (n = 137)	RSV Positive (n = 92)	Total (n = 229)	*p*-Value
Admission				<0.001 *
Admitted	90 (65.7%)	9 (9.8%)	99 (43.2%)	
Discharged home	33 (24.1%)	36 (39.1%)	69 (30.1%)	
Outpatient clinic	14 (10.2%)	47 (51.1%)	61 (26.6%)	
Gender				0.103 *
Female	64 (46.7%)	33 (35.9%)	97 (43.4%)	
Male	73 (53.3%)	59 (64.1%)	132 (57.6%)	
City				<0.001 *
Amman	96 (70.1%)	14 (15.2%)	110 (48.0%)	
Irbid	41 (29.9%)	78 (84.8%)	119 (52.0%)	
Age (months)				<0.001 *
mean (SD)	30.45 ± 18.43	17.47 ± 17.16		
median (IQR)	30.47 (11.88–48.95)	9.45 (5.14–29.32)		

* Chi-square test, statistically significant at *p* < 0.05.

**Table 2:** Symptoms associated with RSV results in the emergency and outpatient departments.

Symptom	RSV Negative (n = 137)	RSV Positive (n = 92)	Total (n = 229)	*p*-Value
Temperature				
Temperature ≥ 38 °C	134 (97.8%)	86 (93.5%)	220 (96.1%)	0.196
Temperature < 35 °C	0 (0.0%)	1 (1.1%)	1 (0.4%)	
Abnormal WBC count/differential	10 (7.3%)	10 (10.9%)	20 (8.7%)	0.348
Tachypnea	43 (31.4%)	19 (20.7%)	62 (27.1%)	0.073
Cyanosis	11 (8.0%)	5 (5.4%)	16 (7.0%)	0.450
Cough	125 (91.2%)	88 (95.7%)	213 (93.0%)	0.199
Pleuritic chest pain	0 (0.0%)	5 (5.4%)	5 (2.2%)	0.006
Hemoptysis	1 (0.7%)	1 (1.1%)	2 (0.9%)	0.776
Dyspnea	4 (2.9%)	8 (8.7%)	12 (5.2%)	0.054
Sore throat	37 (27.0%)	9 (9.8%)	46 (20.1%)	0.001
Runny nose	116 (84.7%)	78 (84.8%)	194 (84.7%)	0.982
Grunting	2 (1.5%)	7 (7.6%)	9 (3.9%)	0.019
Wheeze	54 (39.4%)	67 (72.8%)	121 (52.8%)	<0.001
Difficulty in breathing	20 (14.6%)	31 (33.7%)	51 (22.3%)	0.001
Apnea	4 (2.9%)	1 (1.1%)	5 (2.2%)	0.352
Sputum production	64 (46.7%)	18 (19.6%)	82 (35.8%)	<0.001

**Table 3:** Selected risk factors associated with RSV results in the emergency and outpatient departments.

Variable	RSV Negative (n = 137)	RSV Positive (n = 92)	Total (n = 229)	*p*-Value
Preterm or full term				0.503
Preterm	19 (13.9%)	10 (10.9%)	29 (12.7%)	
Full term	118 (86.1%)	82 (89.1%)	200 (87.3%)	
Delivery				0.191
Caesarean section	64 (46.7%)	35 (38.0%)	99 (43.2%)	
Normal vaginal delivery	71 (51.8%)	57 (64.1%)	128 (55.9%)	
Assisted vaginal (forceps/vacuum)	2 (1.5%)	0 (0.0%)	2 (0.9%)	
Meconium-stained liquor				0.411
No	136 (99.3%)	92 (100.0%)	228 (99.6%)	
Yes	1 (0.7%)	0 (0.0%)	1 (0.4%)	
NICU admission				0.495
No	113 (82.5%)	79 (85.9%)	192 (83.8%)	
Yes	24 (17.5%)	13 (14.1%)	37 (16.2%)	
NICU & ventilation				0.930
No	127 (92.7%)	85 (92.4%)	212 (92.6%)	
Yes	10 (7.3%)	7 (7.6%)	17 (7.4%)	
Surfactant given				0.002
No	110 (80.3%)	87 (94.6%)	197 (86.0%)	
Yes	27 (19.7%)	5 (5.4%)	32 (14.0%)	
Breastfed				0.014
Exclusive	38 (27.7%)	37 (40.2%)	75 (32.8%)	
Mixed	54 (39.4%)	40 (43.5%)	94 (41.0%)	
No	45 (32.8%)	15 (16.3%)	60 (26.2%)	
Special milk/diet				0.064
No	132 (96.4%)	92 (100.0%)	224 (97.8%)	
Yes	5 (3.6%)	0 (0.0%)	5 (2.2%)	
Overcrowding				0.074
No	112 (81.8%)	66 (71.7%)	178 (77.7%)	
Yes	25 (18.2%)	26 (28.3%)	51 (22.3%)	
Siblings <5 years				0.006
No	76 (55.5%)	34 (37.0%)	110 (48.0%)	
Yes	61 (44.5%)	58 (63.0%)	119 (52.0%)	
Parental smoking				0.002
No	2 (1.5%)	10 (10.9%)	12 (5.2%)	
Yes	135 (98.5%)	82 (89.1%)	217 (94.8%)	
Smoking inside home				0.408
No	82 (59.9%)	50 (54.3%)	132 (57.6%)	
Yes	55 (40.1%)	42 (45.7%)	97 (42.4%)	
Mother smoked during pregnancy				0.476
No	131 (95.6%)	86 (93.5%)	217 (94.8%)	
Yes	6 (4.4%)	6 (6.5%)	12 (5.2%)	
Patient on regular meds				0.032
No	111 (81.0%)	84 (91.3%)	195 (85.2%)	
Yes	26 (19.0%)	8 (8.7%)	34 (14.8%)	
Mother’s education				<0.001
Diploma school	15 (10.9%)	8 (8.7%)	23 (10.0%)	
Primary school	4 (2.9%)	23 (25.0%)	27 (11.8%)	
Secondary school	43 (31.4%)	41 (44.6%)	84 (36.7%)	
University (BSc)	54 (39.4%)	19 (20.7%)	73 (31.9%)	
Postgraduate	21 (15.3%)	1 (1.1%)	22 (9.6%)	
Father’s education				<0.001
Diploma school	10 (7.3%)	4 (4.3%)	14 (6.1%)	
Primary school	5 (3.6%)	29 (31.5%)	34 (14.8%)	
Secondary school	51 (37.2%)	42 (45.7%)	93 (40.6%)	
University (BSc)	53 (38.7%)	15 (16.3%)	68 (29.7%)	
Postgraduate	18 (13.1%)	2 (2.2%)	20 (8.7%)	

**Table 4:** Descriptive statistics of clinical findings (Signs) by RSV positivity.

		PCR RSV Result				
		Negative		Positive		
		Count (n = 137)	% Out of Negative	Count (n = 92)	% Out of Positive	*p*-Value *
Chest X-ray infiltrate	Yes	53	38.69%	71	77.17%	<0.001
Blood routine numbers	<4.0	20	14.60%	28	30.43%	<0.001
	4 to 10	10	7.30%	15	16.30%	
	>10.0	23	16.79%	28	30.43%	
	NA	84	61.31%	21	22.83%	
Other clinical manifestations	Yes	42	30.66%	9	9.78%	<0.001
Cardiovascular	Yes	0	0.00%	1	1.09%	0.222
Low activity level	Yes	52	37.96%	26	28.26%	0.130
Apnea > 10 s	Yes	1	0.73%	0	0.00%	0.412
Dehydration	Yes	5	3.65%	1	1.09%	0.230
Hypoxia < 92	Yes	1	0.73%	2	2.17%	0.351
Subcostal/intercostal retractions	Yes	9	6.57%	3	3.26%	
Wheezes	Yes	59	43.07%	68	73.91%	<0.001
Tachypnea	Yes	35	25.55%	13	14.13%	0.044
Cyanosis	Yes	1	0.73%	1	1.09%	0.783
Pneumothorax/atelectasis	Yes	0	0.00%	0	0.00%	
Nasal flaring	Yes	1	0.73%	2	2.17%	0.352
Non-invasive oxygen ventilation was needed	Yes	4	2.92%	1	1.09%	0.350
Invasive oxygen ventilation was needed	Yes	0	0.00%	0	0.00%	
Acidosis	No	7	5.11%	4	4.35%	
	Yes	4	2.92%	4	4.35%	0.821
	NA	126	91.97%	84	91.30%	
Bacterial Co-infection	Yes	2	1.46%	1	1.09%	0.812
Fungal Co-infection	Yes	0	0.00%	1	1.09%	0.220

* Based on Chi-Square analysis.

**Table 5:** Investigating clinical Factors (Symptoms) Associated with RSV-A versus RSV-B results among ED and OP.

RSV A vs. B	RSV_A (n = 17)			RSV_B (n = 75)			
	Mean	Median	Std. Deviation	Mean	Median	Std. Deviation	*p*-Value
Fever days (Clinical)	2.41	2.00	1.326	2.12	2.00	1.174	0.369
Cough days (Clinical)	2.41	2.00	1.326	2.19	2.00	1.363	0.538
Sore throat days (Clinical)	0.12	0.00	0.485	0.41	0.00	1.347	0.376
Runny nose days (Clinical)	2.06	2.00	1.197	2.07	2.00	1.622	0.985
Blocked nose days (Clinical)	0.18	0.00	0.728	0.53	0.00	1.750	0.413
Poor Feeding days (Clinical)	0.65	0.00	1.222	0.27	0.00	0.977	0.171
Hypoxia/Cyanosis days (Clinical)	0.00	0.00	0.000	0.01	0.00	0.115	0.637
Breathlessness days (Clinical)	0.88	0.00	1.166	0.91	0.00	1.472	0.949
Respiratory crackles days (Clinical)	0.94	0.00	1.345	0.27	0.00	1.082	0.029
Apnea > 10 sec days (Clinical)	0.00	0.00	0.000	0.00	0.00	0.000	
Wheezing days (Clinical)	1.76	2.00	1.033	1.49	1.00	1.319	0.429
Low activity level days (Clinical)	0.59	0.00	1.121	0.69	0.00	1.241	0.749
Tachypnea days (Clinical)	0.35	0.00	0.996	0.31	0.00	0.885	0.850
Post Tussive Vomiting days (Clinical)	0.94	0.00	1.345	0.15	0.00	0.711	0.001
Other days (Clinical)	0.47	0.00	1.068	0.21	0.00	0.810	0.269
Max Clinical Symptom Days	2.47	2.00	1.328	2.37	2.00	1.583	0.815

**Table 6:** Binary logistic regression analysis of factors associated with RSV-positive results across all age groups.

Variable	B	*p*-Value	Adjusted OR	95% CI for OR
Admission through		<0.001		
ER without referral	2.725	<0.001	15.262	5.737–40.597
Outpatient clinic without referral	3.760	<0.001	42.931	15.097–122.086
Referred to ED (reference group)			1.00	
Age in months	−0.036	<0.001	0.964	0.947–0.982
Systemic steroid use (Yes vs. No)	−1.835	0.021	0.160	0.034–0.759
Constant	0.088	0.903	1.092	

## Data Availability

The original contributions presented in the study are included in the article/Supplementary Material; further inquiries can be directed to the corresponding author.

## Supplementary Materials

The following supporting information can be downloaded at: https://www.mdpi.com/article/10.3390/v18010133/s1. Table S1: Investigating demographic Factors Associated with RSV A versus B results among Emergency Department and Outpatients.

## Abbreviations

The following abbreviations are used in this manuscript:

RSV	Respiratory Syncytial Virus
ED	Emergency Department
CRF	Case Report Form
VTM	Viral Transport Media
ARIs	Acute Respiratory Infections
