# Supplementary material for: Respiratory Syncytial Virus Positivity Rate and Clinical Characteristics Amongst Children Under 5 Years of Age at the Emergency and Outpatient Settings in Jordan: A Cross-Sectional Study
Source: Viruses. 2026 Jan 20;18(1):133. doi: 10.3390/v18010133 (PMC12846633; doi:10.3390/v18010133)
Supplement: Supplementary file 1 [file viruses-18-00133-s001.zip › Supplementary Table 24 dec.pdf]

Table S1. Investigating demographic Factors Associated with RSV A versus B results among Emergency Department and Outpatients.

			**RSV A VS B**		**Total**	
			RSV- A (n=17)	RSV- B (n=75)		P-value
**Admission through**	ER referral	Count	5	4	9	**0.010**
		% within admission through	55.6%	44.4%	100.0%	
		% within RSV A VS B	29.4%	5.3%	9.8%	
	ER without referral	Count	6	30	36	
		% within admission through	16.7%	83.3%	100.0%	
		% within RSV A VS B	35.3%	40.0%	39.1%	
	Outpatient clinic	Count	6	41	47	
		% within admission through	12.8%	87.2%	100.0%	
		% within RSV A VS B	35.3%	54.7%	51.1%	
**Region**	Middle	Count	6	8	14	**0.011**
		% within Region	42.9%	57.1%	100.0%	
		% within RSV A VS B	35.3%	10.7%	15.2%	
	North	Count	11	67	78	
		% within Region	14.1%	85.9%	100.0%	
		% within RSV A VS B	64.7%	89.3%	84.8%	
**City**	Amman	Count	6	8	14	**0.011**
		% within City	42.9%	57.1%	100.0%	
		% within RSV A VS B	35.3%	10.7%	15.2%	
	Irbid	Count	11	67	78	
		% within City	14.1%	85.9%	100.0%	

	% within RSV A VS B	64.7%	89.3%	84.8%	
**Age**					0.737
**mean, SD**		18.74, 14.94	17.18, 17.70		
**median, Q1-Q3**		9.5, [6.67- 32.62]	9.4, [4.27- 29.17]		
